# Antibacterial Activity of Honey/Chitosan Nanofibers Loaded with Capsaicin and Gold Nanoparticles for Wound Dressing

**DOI:** 10.3390/molecules25204770

**Published:** 2020-10-17

**Authors:** Sharafaldin Al-Musawi, Salim Albukhaty, Hassan Al-Karagoly, Ghassan M. Sulaiman, Mona S. Alwahibi, Yaser Hassan Dewir, Dina A. Soliman, Humaira Rizwana

**Affiliations:** 1 Faculty of Biotechnology, Al-Qasim Green University, Babylon 51013, Iraq; 2 Department of Basic Sciences, College of Nursing, University of Misan, Maysan 62001, Iraq; albukhaty.salim@uomisan.edu.iq; 3 Department of Internal and Preventive Medicine, Veterinary Medicine College, University of Al-Qadisiyah, Al-Diwaniyah 58002, Iraq; alkaragolyh@gmail.com; 4 Department of Applied Sciences, University of Technology, Baghdad 10066, Iraq; 5 Department of Botany and Microbiology, College of Science, King Saud University, P.O. Box 2455, Riyadh 11451, Saudi Arabia; malwhibi@ksu.edu.sa (M.S.A.); dsoliman@ksu.edu.sa (D.A.S.); hrizwana@ksu.edu.sa (H.R.); 6 College of Food and Agriculture Sciences, King Saud University, Riyadh 11451, Saudi Arabia; ydewir@ksu.edu.sa; 7 Faculty of Agriculture, Kafrelsheikh University, Kafr El-Sheikh 33516, Egypt

**Keywords:** honey, chitosan, electrospun, nanofibers, capsaicin, antibacterial, gold nanoparticle, wound dressing

## Abstract

This paper describes the preparation, characterization, and evaluation of honey/tripolyphosphate (TPP)/chitosan (HTCs) nanofibers loaded with capsaicin derived from the natural extract of hot pepper (*Capsicum annuum* *L*.) and loaded with gold nanoparticles (AuNPs) as biocompatible antimicrobial nanofibrous wound bandages in topical skin treatments. The capsaicin and AuNPs were packed within HTCs in HTCs-capsaicin, HTCs-AuNP, and HTCs-AuNPs/capsaicin nanofibrous mats. In vitro antibacterial testing against *Pasteurella multocida, Klebsiella rhinoscleromatis,* *Staphylococcus pyogenes,* and *Vibrio vulnificus* was conducted in comparison with difloxacin and chloramphenicol antibiotics. Cell viability and proliferation of the developed nanofibers were evaluated using an MTT assay. Finally, in vivo study of the wound-closure process was performed on New Zealand white rabbits. The results indicate that HTCs-capsaicin and HTCs-AuNPs are suitable in inhibiting bacterial growth compared with HTCs and HTCs-capsaicin/AuNP nanofibers and antibiotics (*P* < 0.01). The MTT assay demonstrates that the nanofibrous mats increased cell proliferation compared with the untreated control (*P* < 0.01). In vivo results show that the developed mats enhanced the wound-closure rate more effectively than the control samples. The novel nanofibrous wound dressings provide a relatively rapid and efficacious wound-healing ability, making the obtained nanofibers promising candidates for the development of improved bandage materials.

## 1. Introduction

Chronic non-healing wounds present a substantial economic burden on healthcare systems [1]. Among different affecting factors, bacterial contamination has a major influence on wound healing. The design and production of efficient antibacterial dressings for wounds are the subjects of intensive research efforts [2]. Herein, we developed wound dressings that control the liberation of antibacterial agents with effective antibacterial properties while providing acceptable doses to the wound site [3]. 

Noble nanomaterials, such as gold nanoparticles (AuNPs), offer attractive and unique optical properties, such as surface plasmon resonance, which can be used for different detection and therapeutic applications. Several AuNPs have been utilized to improve dressings for the treatment of skin wounds [4]. However, AuNPs can induce cytotoxicity during preparation and in vivo delivery. As such, their potential long-term adverse effects should be carefully considered [5]. A key issue to be resolved before a nanoparticle can be integrated within a biological molecule is its surface chemistry, which determines its stability, functionality, and possible applications [6]. Due to the strong surface reactivity of free electrons, uncoated AuNPs are sensitive to pH, temperature, electrolyte levels, and solvents, and also tend to aggregate when used in such media [7]. To address this issue, various ligand protected AuNPs have been synthesized. Protective species, usually in the form of amine chemical groups, are typically added mostly during synthesis to obtain stable colloids.

Natural and readily available polymers, such as chitosan (Cs), are the most frequently used additives for this purpose [7,8]. Cs is a glycosaminoglycan obtained via the heterogeneous deacetylation of chitin [9,10]. It exhibits a high degree of biocompatibility and low toxicity [11,12], has immune-stimulating properties [13,14], and due to its unique polycationic character, it binds quickly to negatively charged cellular membranes or anionic AuNPs. Moreover, amine chemical groups present in its structure enable chitosan in binding to serum proteins and recognize specific receptors on different cancer cells [14,15]. Chitosan has been extensively applied in wound dressing because of its effective antibacterial properties, water absorption, biocompatibility, biodegradability, non-cytotoxicity, non-antigenicity, and biological membrane function [16].

Wound-healing bio-nanocomposites based on castor oil polymeric films reinforced with chitosan-modified zinc-oxide nanoparticles demonstrate outstanding cytocompatibility and enhanced mechanical properties [17,18]. Although chitosan possesses many useful properties, its applicability in wound dressing has been limited in the past by its low mechanical strength and effective antibacterial activity only in a liquid state. Additionally, its antibacterial activity is insufficient for effective wound dressing [19].

Sarhan et al. reported a honey/chitosan nanofiber with antibacterial, biocompatibility, and moisturizing properties for wound dressing. A combination of chitosan and AuNPs results in innovative materials that can be applied to a wide range of medicinal applications, including wound healing [20,21,22]. Medicinal plants containing a variety of active and effective components, such as flavonoids, essential oils, alkaloids, phenolic compounds, terpenoids, and fatty acids, can provide potent and promising therapeutics for wound healing applications.

Traditional medicines based on such medicinal plants are often preferred over modern therapies due to low costs, few adverse effects, bioavailability, and efficacy [23,24,25,26]. Among different antimicrobial agents, plant extracts have attracted significant attention in antibacterial studies [27,28,29]. Capsaicin (8-Methyl-*N*-vanillyl-6-nonenamide), the active component of cayenne pepper has been grown as a food and for medicinal purposes since ancient times. Multiple pharmacological and physiological properties, such as anti-microbial and anti-virulence activities, are associated with capsaicin and are the subject of ongoing research activities [30,31,32]. The efficacy of active compounds in medicinal plants can be improved by either nanosizing or incorporating them into nanostructures [33,34]. 

Nanomaterials possess unique characteristics due to their size and high surface-area-to-volume ratio. Moreover, nanosized-medicinal plants can occur in association with modifications of their physical and chemical characteristics [35]. Natural based-products can be used directly to treat wounds or as carriers for drug delivery [33]. The efficacy of nanostructured medicinal plants warrants a comprehensive review of both plant-based nanomaterials obtained by different methods and their therapeutic targets in regulating the wound-healing process [33,36]. 

In this study, a novel nanofibrous compound with high levels of honey and chitosan (CS) and enriched with capsaicin and AuNPs was developed for progressive antibacterial and injured-tissue healing efficacy. Honey and chitosan are well-established antibacterial agents that enhance the wound-healing process. The nanofibrous dressing stimulates the extracellular texture of the skin and therefore increases the wound-healing mechanism. This study aims at identifying a method of accelerating the wound-healing process using such materials. We intensified the antibacterial efficacy of honey/tripolyphosphate (TPP)/chitosan (HTCs) mats by enriching them with capsaicin and AuNPs and evaluated their antibacterial properties through in vitro and in vivo study of their wound-healing abilities.

## 2. Materials and Methods

### 2.1. Materials

Capsaicin was obtained from Sigma-Aldrich (St. Louis, MO, USA). Honey was obtained directly from beekeepers (Al-Maimouna, Maysan, Iraq). Dimethyl sulfoxide (DMSO) (≥99.8%), ketamine hydrochloride (≥99%), xylene hydrochloride (≥99%), tri-sodium citrate (≥99%), and gold chloride (≥99.9%) were purchased from Sigma-Aldrich (St. Louis, MO, USA). Chitosan (CS) (≥99%) with a molecular weight of 161 kDa was supplied by HiMedia Company (Mumbai, India). Tripolyphosphate (TPP) (105 kDa) was purchased from Sigma-Aldrich. Glacial acetic acid (99–100% purity) was purchased from Sisco Research Laboratories Pvt. Ltd. (Mumbai, India), and Muller–Hinton agar was prepared by Oxoid Company (London, UK). Aquacel^®^ silver (Ag) was purchased from ConvaTec, Inc. (Deeside, UK). All cell culture materials, such as Dulbecco’s Modified Eagle Medium (DMEM) and fetal bovine serum (FBS) were purchased from Sigma-Aldrich, USA.

### 2.2. Preparation of Honey/Tripolyphosphate (TPP)/Chitosan (HTCs) 

Chitosan nanoparticles were prepared by ionic gelation of TPP with chitosan as reported in the literature [37] with some modifications. Briefly, chitosan powder (5 mg) was dissolved in glacial acetic acid and double-distilled water (DDW) (*v*/*v* = 1:3) at room temperature with continuous stirring for 8 h using a magnetic stirrer. After a clear chitosan solution was obtained. Moreover, 2 mg/mL TPP was dissolved in deionized distilled water separately. Afterward, 20 mL of TPP solution was added dropwise into 50 mL of chitosan solution at ambient temperature with continuous stirring at a speed of 800 rpm. Thereafter, 30 g of honey was diluted in 40 mL of DDW, and the resultant solution was added to the chitosan/TPP suspension.

### 2.3. Synthesis of Gold Nanoparticles (AuNPs)

Initially, 6 mL of 65 mM tetrachloroauric acid (HAuCl4) was added to 500 mL of distilled deionized water and stirred continuously with a magnetic stirrer while being heated to boil on a hot plate. Moreover, 20 mL of 20 mM trisodium citrate was poured into the solution, and the reaction was allowed to continue until the solution turned red. The solution was then cooled to ambient temperature. The AuNPs were separated via centrifugation (14,000 rpm, 7 min) and re-dispersed in 1 mL of phosphate buffered saline (PBS) at room temperature, which was subsequently was stored at 4 °C for further use.

### 2.4. Preparation of Electrospun Suspensions

Different suspensions of HTCs, HTCs-capsaicin, HTCs-AuNPs, and HTCs-capsaicin/AuNPs were prepared. To prepare HTCs-capsaicin, 2 mL of capsaicin was dissolved in DDW (1 mg/mL) to form 50% (*v/v*) of the HTCs-capsaicin solvent. Afterward, 25% (*w/v*), 3% (*w/v*), and 8% (*w/v*) ratios of honey, chitosan, and TPP, respectively, were dissolved in the HTCs-capsaicin solvent. Both suspensions of HTCs and HTCs-capsaicin were enriched with at 10% (*v/v*) AuNPs and stirred for 45 min to produce HTCs-AuNP and HTCs-capsaicin/AuNP suspensions, which were left at ambient condition for 96 h (Table 1).

### 2.5. Viscosity Measurement

The viscosities of the HTCs -capsaicin, HTCs-AuNP, and HTCs-capsaicin/AuNP suspensions were determined and compared with that of the HTCs suspension using a Cannon–Fenske viscometer (Yuhuan Ocean Biochemical Co. Ltd., Taizhou, China) at a temperature and stirring speed of 28 °C and 6 rpm, respectively.

### 2.6. Electrospinning Process

The electrospinning setup consists of a syringe pump (PHD 200 Infusion, Harvard Apparatus, Holliston, MA, USA), stainless-steel spinneret needle (21 G × 1.5, Lexington, KY 40505, USA), high-voltage power supply (14-20000, FuG Electronik GmbH, Berlin, Germany), 10 cm × 10 cm collector, and 2 mL syringe. The distance between the needle and the collector was kept at 20 cm, and an applied voltage of 25 kV was used. Flow rates for all suspensions were set to 0.4 mL/h. All acquired nanofibrous mats were produced for 4 h. Due to the presence of capsaicin in the HTCs and HTCs-AuNP suspensions, electrospinning parameters were changed to a voltage of 28 kV and a flow rate of 0.7 mL/h. Due to the difficulty of electrospinning the suspensions, a distance of 15 cm was maintained between the collector and the needle.

### 2.7. Characterization and Measurements

Morphological characterizations of the prepared HTCs, HTCs-capsaicin, HTCs-AuNP, and HTCs-capsaicin/AuNP mats were estimated and observed via field emission-scanning electron microscopy (FE-SEM; Supra 35 VP, Carl Zeiss, Germany). The diameters of the nanofibers were evaluated using the ImageJ software (Caliper Life Sciences, Hopkinton, MA, USA). One hundred fibers from each fabricated nanofibrous mat were measured in three images, and the average size was estimated.

### 2.8. Antibacterial Study

The antibacterial activities of the developed HTCs, HTCs-capsaicin, HTCs-AuNP, and HTCs-capsaicin/AuNP mats were estimated and compared with those of difloxacin and chloramphenicol antibiotics, which served as the positive control. Blend suspensions were evaluated to determine their antibacterial activities against *Pasteurella multocida, Klebsiella rhinoscleromatis, Staphylococcus pyogenes,* and *Vibrio vulnificus.* Each tested bacterial specie was cultured on a separate Petri dish (MatTek, Ashland, MA, USA) containing Muller–Hinton agar (Qiagen, Mississauga, ON, Canada). Three wells were made in each dish, into which 0.5 mL of each blend suspension was injected. Afterward, the Petri dishes were incubated for 48 h at 37 °C. After the incubation, the inhibition zone was measured and compared with that of the antibiotic materials.

### 2.9. Cytotoxicity Assay (MTT Assay)

HTCs, HTCs-capsaicin, HTCs-AuNP, and HTCs-capsaicin/AuNP mats, along with positive control of Aquacel^®^ Ag, were assayed to determine their toxicity. The nanofibrous mats were disinfected with ultraviolet (UV) light for 20 min and immersed in DMEM (containing FBS). Afterward, the obtained extract suspensions of nanofibrous mats and Aquacel^®^ Ag were incubated at 37 °C and the extracted suspensions were filtered through sterile filters (0.22 µm, Sigma, USA). The DMEM medium was used to prepare different dilution ratios (0, 25, 50, and 100%) of the extract. Vero cells (fibroblasts) from the kidneys of an African green (or old world) monkey (*Cercopithecus aethiops*) (1 × 10^4^ cells/well) were incubated for 24 h at 37 °C in a 96-well plate. The Vero cells were then incubated with the extracted material at different dilution ratios for 48 h. Next, 20 µL of MTT solution was added to each well, and the resultant mixture was incubated for 4 h. Subsequently, DMSO (100 µL) was added to each well to dissolve the formazan crystals. The optical density (OD) was evaluated at a wavelength of 590 nm to calculate the effect of each sample on cell viability.

### 2.10. Vero Cell Proliferation

The cellular proliferative efficacy of the prepared HTCs, HTCs-capsaicin, HTCs-AuNP, HTCs-capsaicin/AuNP mats, and Aquacel^®^ Ag (as the positive control) was determined. Vero cells were seeded (1 × 10^4^ cells/well) on the examined samples and incubated differently for 24 and 72 h. At each time point, the estimated samples were transferred from the primary plate to a new plate with fresh media and MTT solution (100 µL/well) and incubated for 4 h. DMSO was used to dissolve the obtained formazan crystals, and the OD was calculated at 590 nm.

### 2.11. In Vivo Study

Thirty white New Zealand rabbits (2.5 kg/animal) were used as models to study the in vivo wound-healing process. The animals were housed in cages with dimensions of 140 cm × 120 cm × 90 cm under a 12 h:12 h light-dark cycle at 25 ± 2 °C and 65% relative humidity with standard granulated food and water available ad libitum. All procedures were performed following the U.S. National Institutes of Health (NIH) Guide for the Care and Use of Laboratory Animals (NIH Publication No. 86–23, revised in 1996) and were approved by the Animal Care and Ethics Committee at Biotechnology Division, Applied Sciences Department, University of Technology, Baghdad, Iraq.

All rabbits were anesthetized with a mixture of ketamine HCl (50 mg/kg) and xylene HCl (20 mg/kg). The back of each rabbit was shaved and a 1-cm wound was made on it back using a biopsy puncher. According to the different treatment methods employed, the experimental groups were divided into 6 groups. HTCs, HTCs-capsaicin, HTCs-AuNP, HTCs-capsaicin/AuNP nanofibrous mats, and Aquacel^®^ Ag were placed on the wound site after UV sterilized for 20 min. Untreated rabbits served as negative controls. The wounds were covered with cotton gauze afterward. Changes in wound areas were measured after 1, 3, 5, 7, 10, and 14 days by observing the change in wound contraction (cm^2^), change in wound color (chromatic red), and luminance in red, green, and blue color values. Each treatment and control were evaluated on four rabbits, and the mean value of the four measurements was recorded by

wound size (%) = [W _(1,3,5,7,10,14)_/W _(0)_] × 100 (1)

where W _(0)_ and W _(1,3,5,7,10,14)_ represent the exposed areas of the wounds on days 0 and 1, 3, 5, 7, 10, and 14, respectively.

### 2.12. Statistical Analysis

The data collected are represented as the mean ± standard deviation (SD) of at least three experiments. Statistical tests were performed utilizing a one-way analysis of variance to compare results. *P* values < 0.01 were considered statistically significant. The statistical analysis was conducted using SPSS v.20 software.

## 3. Results and Discussion

### 3.1. Viscosity Measurements of the HTCs, HTCs-capsaicin, HTCs-AuNP, and HTCs-Capsaicin/AuNP Blend Suspensions

The viscosity of the HTCs, HTCs-capsaicin, HTCs-AuNP, and HTCs-capsaicin/AuNP blend suspensions was evaluated immediately after preparation (nearly 2 h after preparation) and after one week of aging (168 h after preparation). As shown in Table 2, the electrospun HTCs, HTCs-capsaicin, HTCs-AuNP, and HTCs-capsaicin/AuNP nanofibers resulted in significantly higher viscosities immediately after preparation compared with that observed after aging for a week at room temperature. The viscosity was lower after adding capsaicin, AuNPs, and capsaicin/AuNPs to HTCs. The viscosity of HTCs-capsaicin/AuNPs was lower than that of the other three blend suspensions.

In this study, HTCs-based dressings were developed. The antibacterial activity of a developed dressing alone is reportedly insufficient for a complete wound-healing process. Other elements are required to increase the wound-healing percentage [38,39]. The main advantages of the developed dressing are remarkable antibacterial properties as well as significant and rapid wound-closure capacity [40,41]. As shown in Table 2, HTCs suspension enriched with capsaicin and AuNPs resulted in a noticeable reduction in viscosity. The AuNPs, therefore, had a stronger effect on the decrease in viscosity compared with capsaicin. A decrease in the viscosity of HTC-capsaicin/AuNPs mats in contrast to HTCs-capsaicin and HTCs-AuNP mats could enhance the antibacterial effect [42]. However, further studies are required to determine the primary cause of the reduction. Similar observations of viscosity were reported by Sarhan et al. [22].

### 3.2. Electrospinning of the HTCs, HTCs-Capsaicin, HTCs-AuNP, and HTCs-Capsaicin/AuNP Suspensions

Electrospun HTCs nanofibers were developed using about 50 *w/v* honey and 5.5 *w/v* chitosan. Among the effective types of developed dressings, the HTCs dressing was composed of remarkable concentrations of chitosan and honey with biocompatible solvents. The capsaicin substituted 50% of the HTCs solvent, whereas the AuNPs enriched the suspensions of the HTCs and HTCs-capsaicin before electrospinning. The obtained blend suspensions were then electrospun and collected as nanofibrous mats for subsequent experiments.

### 3.3. FE-SEM Results

**Figure 1:**
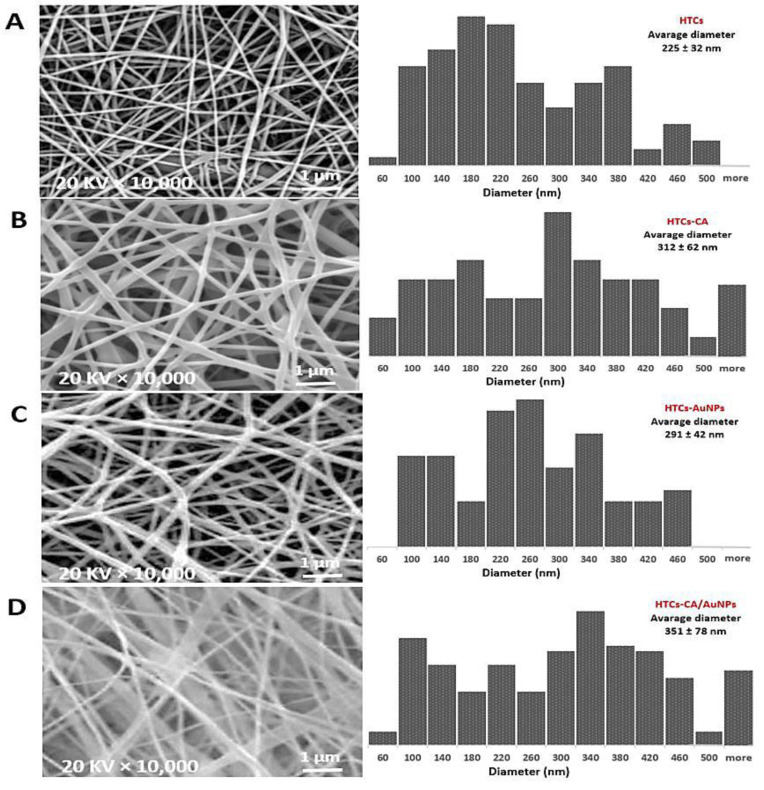
SEM images of the nanofibers mats and different diameter distribution represent changes in morphology and nanofiber diameter of the HTCs mat due to its enriching with the capsaicin, AuNPs and their combination (capsaicin/AuNPs). (**A**) HTCs, (**B**) HTCs-capsaicin, (**C**) HTCs-AuNPs and (**D**) HTCs-capsaicin/AuNPs. (CA refers to capsaicin).

The FE-SEM image of HTCs and HTCs-AuNP nanofibers revealed an identical dense, smooth, clear, and bead-free morphology. Furthermore, adding AuNPs enhanced the nanofiber diameter compared with the other nanofibrous mats. The size of the HTCs nanofibers was enhanced from 225 ± 32 to 291 ± 42 nm due to presence of AuNPs. In the case of HTCs-capsaicin and HTCs-capsaicin/AuNP nanofibers, a uniform deposition was critical. Figure 1 depicts the bimodal distribution of the diameters of the HTCs-capsaicin and HTCs-capsaicin/AuNPs nanofibers. The fibers in the HTCs-capsaicin and HTCs-capsaicin/AuNP mats were not as compact and dense as those in the HTCs and HTCs-AuNP mats. The nanofibers of the HTCs-capsaicin and HTCs-capsaicin/AuNP mats were 312 ± 62 and 351 ± 78 nm in diameter, respectively.

The developed nanofiber mats were successfully tested in vitro and in vivo. From the SEM results, a uniform and bead-free morphology for the HTCs, HTCs-AuNP, HTCs-capsaicin, and HTCs- capsaicin/AuNP mats were observed. The size of the fiber diameter of the HTCs-AuNP, HTCs-capsaicin, and HTCs-capsaicin/AuNP formulations was enhanced compared with that of HTCs enriched with AuNPs and capsaicin alone and together. These results confirm that AuNPs and capsaicin had an incremental effect on the fiber diameter size. Moreover, the SEM micrograph shows that the AuNPs created a denser and compact nanofiber compared with capsaicin. This result is in excellent agreement with previous studies [22,43,44].

### 3.4. Assessment of Antibacterial Activity

**Figure 2:**
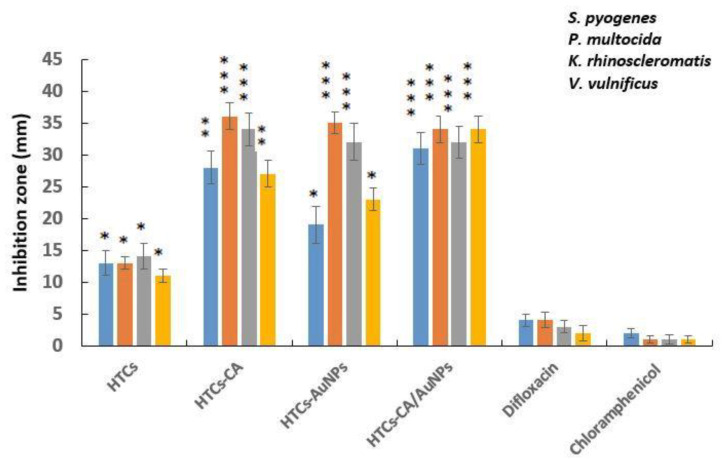
The antibacterial activity of the electrospun mats of HTCs, HTCs-capsaicin, HTCs-AuNPs and HTCs-capsaicin/AuNPs against *P. multocida, K. rhinoscleromatis, S. pyogenes,* and *V. vulnificus*. (Data are presented as mean ± S.D., (* *P* < 0.05; ** *P* < 0.01; *** *P* < 0.001); n = 3). (CA refers to capsaicin).

Resistant bacterial strains is the major challenge affecting chronic non-healing wounds. Evaluation of the antibacterial efficiency confirmed that HTCs nanofibrous mats exhibited slight antibacterial efficiency against all bacterial strains used in this study. HTCs mats showed the same antibacterial efficacy as that of difloxacin antibiotics. To improve antibacterial efficacy, capsaicin and AuNPs and a combination of the two enriched HTCs nanofibers and the products were evaluated for their antibacterial efficacy against *P. multocida, K. rhinoscleromatis, S. pyogenes,* and *V. vulnificus*. The antibacterial effect of difloxacin and chloramphenicol antibiotics was estimated and compared with that of the designed nanofiber mats. Figure 2 shows the antibacterial efficiency of the developed HTCs, HTCs-capsaicin, HTCs-AuNP, and HTCs-capsaicin/AuNP nanofibrous mats against that of difloxacin and chloramphenicol, which served as the positive control. The HTCs-capsaicin and HTCs-AuNP nanofibrous mats exhibited superior antibacterial efficacy against *P. multocida and K. rhinoscleromatis*. HTC-capsaicin/AuNP mats were the most effective against *S. pyogenes* and *V. vulnificus* (Figure 2). Such superior performance is mainly due to the enrichment of HTCs with capsaicin and AuNPs. The results also reveal the antibacterial activity of HTCs-capsaicin/AuNP mats against all used bacterial strains. Moreover, a lower antibacterial effect of HTC-AuNP mats against *S. pyogenes,* and *V. vulnificus* were observed in comparison with that of HTC-capsaicin (Figure 2). HTCs-capsaicin/AuNP and HTCs-AuNP mats exhibited the same antibacterial activity against *K. rhinoscleromatis*.

The antibacterial properties of honey are related to the level of hydrogen peroxide and other non-peroxide factors as lysozyme, phenolic acids, and flavonoids [45,46]. The polycationic nature of Chitosan is a major cause of its antibacterial activity, which leads to cell leakage and death due to the interaction with the negatively charged surfaces of the bacteria [47]. AuNPs can directly induce bacterial cell wall damage, acting as bactericidal and bacteriostatic agents as they attach to the bacterial DNA and inhibit the uncoiling of the double-helix structure during replication or transcription [48]. Consequently, they can inhibit multidrug-resistant pathogens, such as *P. multocida* and *V. vulnificus*. Furthermore, AuNPs inhibit the formation of reactive oxygen species and therefore behave as antioxidant agents that can aid the healing process [49,50]. The antimicrobial mechanism of capsaicin is related to changes in the cell membrane fluidity due to the disruption of bacterial cell membranes [32]. Antibacterial data show the enhanced antibacterial activity of HTCs-AuNPs against *P. multocida* and *K. rhinoscleromatis* and relatively less antibacterial activity against *S. pyogenes* and *V. vulnificus* (Figure 2). HTCs-capsaicin exhibits high antibacterial efficacy against all bacterial strains. The antibacterial efficiency of honey, chitosan, AuNPs, and capsaicin have been confirmed in Gram-positive and-negative bacteria [51]. Moreover, the increased antibacterial efficiency against *V. vulnificus* compared with *S. pyogenes* has been emphasized in previous studies for chitosan [52] as well as capsaicin extract and a mixture of honey and AuNPs [53]. The data obtained are consistent with the results achieved here concerning the enhanced antibacterial efficacy exhibited against *V. vulnificus* and *P. multocida* strains [54]. Efren et al. (2016) tested poly(ε-caprolactone) (PCL)-formulated silver nanofibrous mats (PCL-AgNPs) on different types of bacterial strains, such as *S. pyogenes* and *V. vulnificus, P. multocida*, *Streptococcus pyogenes,* and *Klebsiella pneumoniae* [55]. They reported a significant antibacterial activity of PCL-AgNP mats on all mentioned strains [54]. In another study, Thenmozhi et al. studied the inhibitory effect of chitosan and honey on *S. pyogenes* and *Staphylococcus aureus*. Their results indicated an inhibitory effect of chitosan and honey on both strains and matches antibacterial data concerning the developed HTCs mats [56].

### 3.5. Cytotoxicity Assay (MTT Assay)

**Figure 3:**
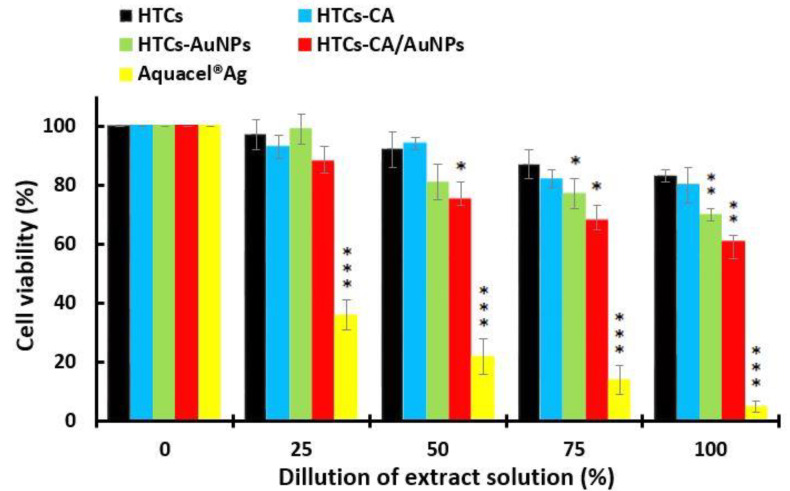
MTT assay for determining the cell viability of the developed nanofibrous mats: HTCs, HTCs-capsaicin, HTCs-AuNPs and HTCs-capsaicin/AuNPs. Aquacel^®^ Ag was the positive control an in both test. (Data are presented as mean ± S.D., (* *P* < 0.05; ** *P* < 0.01; *** *P* < 0.001); n = 3. (CA refers to capsaicin).

The cytotoxicity of the developed HTCs, HTCs-capsaicin, HTCs-AuNP, and HTCs-capsaicin/AuNP nanofibrous mats, and Aquacel^®^ Ag, was evaluated via MTT tests. Cultured Vero cells were subjected to various doses of the extract suspension of all the developed mats (25, 50, 75, and 100%). The cytotoxicity index was then calculated by evaluating the viable cell value after 48 h (Figure 3). The HTCs and HTCs-capsaicin mats showed the highest cell viability rates, at 83 and 80%, respectively, in the treatment of 100% extract suspension. Conversely, the HTCs-AuNP and HTCs-capsaicin/AuNP mats were associated with significantly lower cell viabilities of 70 and 61% (*P* < 0.01), respectively (Figure 3). The commercial wound dressing of Aquacel^®^ Ag showed noticeable cytotoxicity at all mentioned dilutions (*P* < 0.001) (Figure 3). A cell viability assay was conducted by treating the Vero cells with the developed HTCs, HTCs-capsaicin, HTCs-AuNP, and HTCs-capsaicin/AuNP nanofibrous mats in addition to Aquacel^®^ Ag dressing using an MTT test. Most levels of proliferation and cell viability are related to HTCs and HTCs-capsaicin. The addition of the capsaicin extract to HTCs-AuNP nanofibers reportedly increases their viability and cell proliferation levels [22,57,58].

Multiple studies have reported that the modification of chitosan/arginine/AuNPs improved the hydrophilicity, mechanical strength, and antibacterial properties of the film, which in turn provided an enhanced environment for cell adhesion and proliferation. A cell counting Kit-8 (CCK-8) was used to determine the survival rates [59,60].

### 3.6. Vero Cells Proliferation

**Figure 4:**
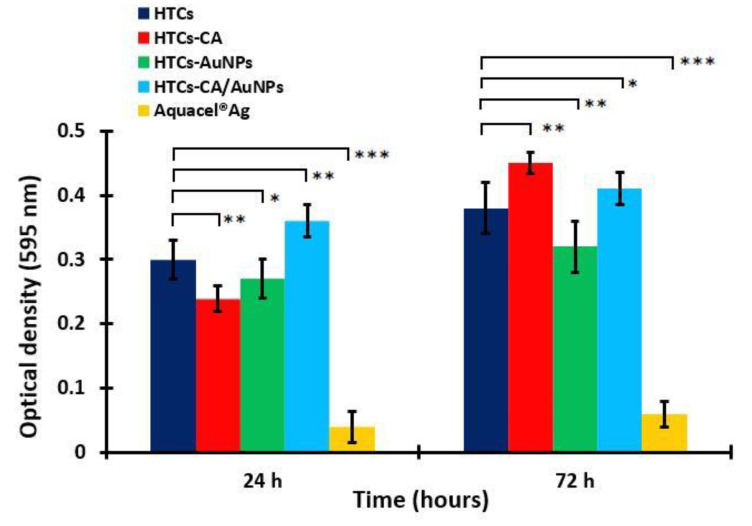
Cell proliferation of the developed nanofibrous mats: HTC, HTC-CA, HTC-AuNPs and HTC-CA/AuNPs. Aquacel^®^ Ag was the positive control an in both test. (Data are presented as mean ± S.D., (* *P* < 0.05; ** *P* < 0.01; *** *P* < 0.001); n = 3. (CA refers to capsaicin).

A cell proliferation study was performed using the treatment of the Vero cells on the HTCs, HTCs- capsaicin, HTCs-AuNP, and HTCs-capsaicin/AuNP nanofibers and Aquacel^®^ Ag for 24 and 72 h (Figure 4) and an MTT assay. The proliferation values of the HTCs, HTCs-capsaicin, and HTCs-capsaicin/AuNP nanofibrous mats increased with culture time. HTCs-capsaicin and HTCs-capsaicin/AuNP mats were associated with the most significant increase in proliferation (*P* < 0.01) after 72 h of incubation (Figure 4), whereas the HTCs-AuNP mats achieved nearly the same proliferation ratio at 24 and 72 h. Aquacel^®^ Ag showed a remarkable cytotoxic effect on cell proliferation, as indicated by its low OD value (Figure 4). The obtained results confirm the similarity in the cytotoxic effect of Aquacel^®^ Ag (Figure 3). All nanofibrous mats show increased cell viability and proliferation compared with Aquacel^®^ Ag (Figure 3 and 4). Previous studies presented various nanofiber dressing mats with no cytotoxic effect on Vero cells, but herein, the developed nanofibrous dressing mats, in addition to its biocompatibility properties, produce a proliferative effect [61,62]. For the wound-healing process, the addition of honey increased tissue recovery by inducing cytokines released from leukocytes and protecting the immune system against infection [37,63]. Chitosan caused the prompt emigration of the polymorph nuclear and mononuclear cells and subsequently degraded into its low-molecular-weight oligomers and monomers. This process led to cell migration and indirectly increased cell proliferation in vivo. Capsaicin reportedly induces osmotic stress and promotes key genes associated with cell-membrane biosynthesis [56].

### 3.7. Assessment of the Wound-Healing Ability

**Figure 5:**
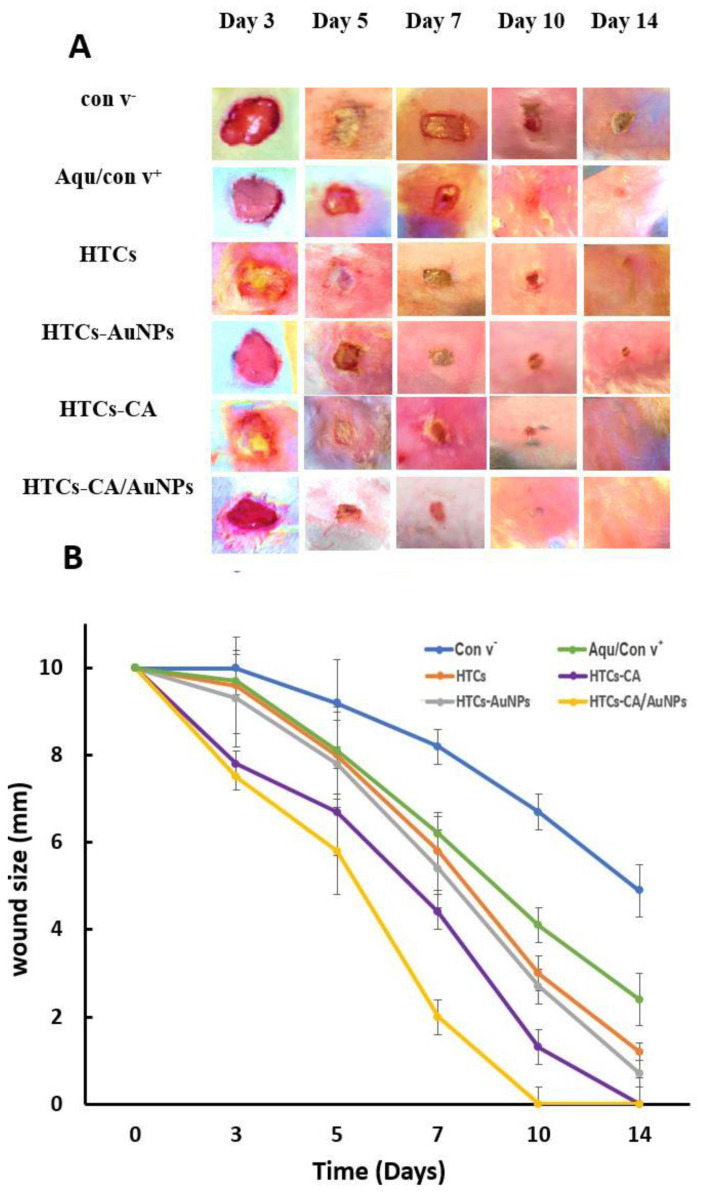
Photographic pictures of wound healing process (**A**) Changes curve of the wound size (**B**) on days 0, 3, 5, 7, 10, and 14 for the all dressings HTCs, HTCs-capsaicin, HTCs-AuNPs and HTCs-capsaicin/AuNPs in addition to the negative (con v-) and positive (Aquacel^®^ Ag) control. (CA refers to capsaicin).

The HTCs, HTCs-capsaicin, HTCs-AuNP, and HTCs-capsaicin/AuNP and Aquacel^®^ Ag dressings were used on a sectional-1 cm wound created on the upper back of the rabbit samples. Wound images were collected on days 0, 3, 5, 7, 10, and 14 to study the changes in wound volume (Figure 5). Figure 5 shows that the wounds with HTCs-capsaicin and HTCs-capsaicin/AuNP dressings decreased in size by day 3 compared with the negative control wounds. A significant decrease in wound size was obtained in all the examined dressings in addition to Aquacel^®^ Ag, compared with that in the negative control on days 5 and 7 (Figure 5). Notably, all prepared nanofibrous dressings stuck easily to the wounds without the need for a biological adhesive. This performance was probably due to the hydrophilic properties of the TTP, chitosan, and honey and the remarkable water solubility of honey. The dressings, therefore, produced an eligible hydrated situation for the wounds. However, Figure 4 shows that all the developed nanofibrous dressings had a vigorous wound-healing ability. The results demonstrate a remarkable rapid tissue-repairing ability in all the developed nanofibrous dressings. Wound healing was noticeably increased with HTCs nanofibrous mats after the addition of capsaicin to HTCs-capsaicin mats. Wound healing was also enhanced after enriching HTCs with AuNPs but at a lower level than that of HTCs-capsaicin. The HTCs enriched with capsaicin and AuNPs produced superior wound-healing rates compared with HTCs-capsaicin and HTCs-AuNP dressings (Figure 4). El-Kased et al., in their study, successfully enhanced the wound-healing process in mice using a honey-chitosan suspension. However, unlike our results, the suspension they obtained was unable to achieve full wound closure after 10 days [64,65]. Additionally, Sarhan et al. studied the effects of a honey-chitosan-based mat on the wound-closure process in animals. Their results showed similar wound-healing effects of the honey-and-chitosan combination using a nanofiber mat [22]. In our study, the developed nanofibrous mats enhanced the total closure of all treated mice wounds at similar times. HTCs enriched with capsaicin and AuNPs separately and in combination achieved an increase in wound-closure effect compared with the commercial Aquacel^®^ Ag. In summary, the novel nanofibrous wound dressings exhibit faster and more efficacious wound-healing abilities compared with many other nanofibrous types of wound dressings. An overview of the key features of the inherent properties of chitosan, its modification, and its use in biomedical engineering, particularly toward anti-inflammatory effects and wound-healing, has been published [66]. Besides, honey–polyvinyl alcohol–chitosan nanofibers reportedly showed pronounced antibacterial activity against *S. aureus* but weak antibacterial activity against *Escherichia coli*. The developed HP-chitosan nanofibers showed no cytotoxic effects on cultured fibroblasts [67].

## 4. Conclusions

Honey, chitosan, and TPP-based nanofibers; nanofibers with 50% of the solvent substituted with capsaicin; and HTCs and HTCs-capsaicin nanofibers loaded with 10% AuNPs in the HTCs-AuNPs and HTCs-capsaicin/AuNPs were prepared via electrospinning. All developed wound dressings and commercial Aquacel^®^ Ag were characterized and evaluated to determine their antibacterial activity, cell viability, proliferation effect, and wound-closure ability. Enriching HTCs with capsaicin and AuNPs separately and in combination significantly reduced the viscosity. The HTCs-capsaicin and HTC-AuNP suspensions showed similar results against *P. multocida* and *K. rhinoscleromatis* bacteria, while the HTCs-capsaicin/AuNP suspension had less effect on such bacteria. The HTCs suspension showed results similar to those of difloxacin with a significantly reduced inhibition zone in comparison with the other suspensions. The HTCs-capsaicin suspension achieved the best results against *V. vulnificus* and *S. pyogenes* bacteria. Furthermore, HTCs, HTCs-capsaicin, and HTCs-capsaicin/AuNP mats exhibited high levels of cell viability and proliferation compared with Aquacel^®^ Ag. The wound-closure ability of all fabricated dressings, except that of HTCs, was stronger than that of Aquacel^®^ Ag. The wound-healing rate of HTCs was similar to that of Aquacel^®^ Ag. Among all the developed dressings and Aquacel^®^ Ag, HTCs-capsaicin mats showed the highest wound-healing rate. The demonstrated effectiveness of the developed dressings, as measured by antibacterial efficiency, cell viability, proliferation effect, and wound-closure ability, along with their negligible side effects, make them promising and competitive candidates for application in efficacious wound dressings.

## Figures and Tables

**Table 1:** Electrospinning parameters used to produce the electrospun fibers.

Sample	Voltage KV	Distance (cm)	Flow (mL^●^h^−1^)
**HTCs**	28	15	0.7
**HTCs-CA**	25	20	0.4
**HTCs-AuNPs**	28	15	0.7
**HTCs-CA/AuNPs**	25	20	0.4

**Table 2:** Change in the viscosity (mPa·s) of the HTCs, HTCs-capsaicin, HTCs-AuNPs and HTCs-capsaicin/AuNPs upon aging.

Sample	2 h	168 h
**HTCs**	120 ± 4.20	111 ± 6.46 *
**HTCs-CA**	101 ± 2.52	81 ± 7.38 **
**HTCs-AuNPs**	76 ± 3.24	49 ± 4.82 **
**HTCs-CA/AuNPs**	60 ± 5.46	38 ± 3.26 ***

(Data are presented as mean ± S.D., (* *P* < 0.05; ** *P* < 0.01; *** *P* < 0.001); n = 3). (CA refers to capsaicin).
